# Dexamethasone-Induced Sarcopenia and Physical Frailty in Children With Acute Lymphoblastic Leukemia: Protocol for a Prospective Cohort Study

**DOI:** 10.2196/33517

**Published:** 2022-04-11

**Authors:** Emma Jacobine Verwaaijen, Annelienke van Hulst, Marta Fiocco, Annelies Hartman, Martha Grootenhuis, Saskia Pluijm, Rob Pieters, Erica van den Akker, Marry M van den Heuvel-Eibrink

**Affiliations:** 1 Princess Máxima Center for Pediatric Oncology Utrecht Netherlands; 2 Department of Pediatric Physiotherapy Erasmus Medical Center-Sophia Children’s Hospital Rotterdam Netherlands; 3 Department of Endocrinology Erasmus Medical Center-Sophia Children’s Hospital Rotterdam Netherlands

**Keywords:** acute lymphoblastic leukemia, sarcopenia, physical frailty, muscle atrophy, dexamethasone, fatigue, physical activity, glucocorticoid-induced atrophy

## Abstract

**Background:**

During treatment for pediatric acute lymphoblastic leukemia (ALL), children receive high doses of dexamethasone for its apoptotic effect on leukemia cells; however, muscle atrophy is a well-known serious side effect. Muscle atrophy (loss of muscle mass) accompanied by a decreased muscle strength may lead to a generalized impaired skeletal muscle state called sarcopenia. Loss of muscle mass is also an indicator of physical frailty, which is defined as a state of increased vulnerability that is characterized by co-occurrence of low muscle mass, muscle weakness, fatigue, slow walking speed, and low physical activity. Both sarcopenia and physical frailty are related to an increased risk of infections, hospitalizations, and decreased survival in children with chronic diseases.

**Objective:**

This study aims to (1) estimate the occurrence of sarcopenia and physical frailty in children during ALL maintenance therapy, (2) evaluate the effect of administering dexamethasone, and (3) explore determinants associated with these outcomes.

**Methods:**

This prospective study is being pursued within the framework of the DexaDays-2 study: a randomized controlled trial on neurobehavioral side effects in pediatric patients with ALL. A total of 105 children (3-18 years) undergoing ALL maintenance treatment at the Princess Máxima Center for Pediatric Oncology are included in this study. Sarcopenia/frailty assessments are performed before and just after a 5-day dexamethasone course. A subset of 50 children participating in the DexaDays-2 trial because of severe dexamethasone-induced neurobehavioral problems were assessed at 3 additional timepoints. The sarcopenia/frailty assessment consists of bioimpedance analysis (skeletal muscle mass [SMM]), handheld dynamometry (handgrip strength), Pediatric Quality of Life Inventory Multidimensional Fatigue Scale (fatigue), Timed Up and Go Test (TUG; walking speed), and physical activity questionnaires. To evaluate potential change in sarcopenia/frailty components after a 5-day dexamethasone administration, a paired Student t test or Mann-Whitney U test will be used. Because of the presence of repeated measurements, generalized linear mixed models will be used to estimate the effect of dexamethasone on sarcopenia and frailty outcomes. Multivariable regression models will be estimated to investigate associations between the assessment scores and patient and treatment-related factors.

**Results:**

Patient accrual started in 2018 and was finalized in spring 2021. From autumn 2021 onward final data analyses will be performed.

**Conclusions:**

This first study combining parameters of sarcopenia and physical frailty is of importance because these conditions can seriously complicate continuation of ALL therapy, independence in physical functioning, reaching motor milestones, and participating in daily life activities. The results will provide knowledge about these complications, the association between dexamethasone treatment and muscle loss and other components of frailty, and therefore insights into the severity of this side effect. By exploring potential determinants that may be associated with sarcopenia and physical frailty, we may be able to identify children at risk at an earlier stage and provide timely interventions.

**International Registered Report Identifier (IRRID):**

DERR1-10.2196/33517

## Introduction

Acute lymphoblastic leukemia (ALL) is the most prevalent pediatric cancer worldwide. Advances in treatment strategies and supportive care have resulted in a 5-year survival rate of about 90% in high-income countries [1-3]. Consequently, there is growing attention for adverse health effects, including impairments in physical performance during and after therapy [4-6]. Impairments in physical performance, either transient or permanent, in children with ALL are usually explained by a neurological disorder, fractures, osteonecrosis, general malaise, pain, or severe muscle atrophy [7-11]. Muscle atrophy, in turn, can be caused by malnutrition, inflammation, low physical activity, and can be aggravated by treatment with glucocorticoids [12,13]. Glucocorticoids, which are essential in the treatment of ALL, are known to regulate protein metabolism in skeletal muscle, thereby inducing a catabolic effect and consequent muscle atrophy [14,15].

Dexamethasone is the most potent glucocorticoid and a cornerstone for the treatment of pediatric ALL, because it reduces the frequency of central nervous system relapse [15]. As it is administered in high doses for considerable periods in various ALL protocols worldwide (6 mg/m^2^ per day) [16-18], children with ALL carry an increased risk of glucocorticoid-induced muscle atrophy [15].

Muscle atrophy (loss of muscle mass) when accompanied by decreased muscle strength may indicate a generalized skeletal muscle disorder called sarcopenia [19]. Sarcopenia, defined as the combination of low muscle mass and strength or function, is associated with increased adverse health outcomes in various adult populations [20]. The presence of sarcopenia has been investigated to a limited extent in 3 previous studies including children with ALL [4,21,22], which indicated that muscle mass loss in children during ALL therapy was associated with the number and duration of hospital admissions [4], occurrence of invasive fungal infections, other adverse events of Common Terminology Criteria for Adverse Events (CTCAE) grade ≥III [21], and even—when fat mass and body mass index were increased—with impaired survival [22]. However, due to small sample sizes and methodological limitations, which make it difficult to correct for relevant confounders, it is unclear if these results indicate a true causal relationship or coassociation.

Physical frailty is another undesired consequential state, and is characterized by 5 components: unintentional weight loss (due to muscle mass loss), muscle weakness, self-reported exhaustion, slow walking speed, and low physical activity [23]. Physical frailty has been reported as a state of reduced physiologic reserve with increased vulnerability to stressors. It was first defined in the elderly by Fried et al [23], and was shown to be associated with disabilities and early mortality in young adult survivors of childhood cancer [24-27]. The 5 components of physical frailty have all been individually described to occur in children with ALL. For example, higher levels of fatigue were reported in children with ALL compared with children from the general population, and more often during dexamethasone treatment [28,29]. Besides, several studies showed that children with ALL had muscle mass loss [22], muscle weakness [5,6,30], slow walking speed [5,31], and reduced physical activity levels [32-34]. It is therefore relevant to study whether co-occurrence of these 5 physical frailty components may be prevalent in children with ALL, putting them at risk for serious complications.

There is some known overlap between physical frailty and sarcopenia; in fact, sarcopenia has been reported as a precursor of frailty in older adults [23,35-37]. The biological and clinical relationships between these 2 states in pediatric cancer populations are not clear yet.

The development of sarcopenia or physical frailty or both in children during ALL therapy is undesirable because of the consequences it may have for therapy (discontinuation or dose reduction due to clinical state), physical abilities, motor development, and child participation levels in daily life activities, as well as the potential negative effects on the longer term. To our knowledge, physical frailty in children has been assessed in only 2 previous studies, but not yet in pediatric patients with cancer. In both studies the frailty phenotype was associated with severe infections and increased hospitalizations [38,39].

So far, frailty has not been examined in children during ALL treatment nor has the relationship between dexamethasone and sarcopenia/frailty been investigated. Hence, the aims of this study are to estimate the occurrence of sarcopenia and physical frailty in children with ALL during maintenance therapy, to evaluate the effect of administering dexamethasone on sarcopenia and frailty (and their individual components) and to explore potential determinants associated with these outcomes. This paper describes the statistical design and methodology for this study.

## Methods

### Study Design and Patient Recruitment

This prospective national observational cohort study is taking place at the Princess Máxima Center for Pediatric Oncology, Utrecht, the Netherlands. The children included in this study are participating in the DexaDays-2 study: a randomized controlled trial on neurobehavioral side effects in pediatric patients with ALL [40]. In that study, Dutch patients with ALL are eligible when they fulfill the following criteria: age 3-18, confirmed diagnosis of ALL, and inclusion in the Dutch Childhood Oncology Group (DCOG) medium-risk group of the ALL-11 protocol. Only patients between 3 and 18 years can participate as this was an inclusion criterion for the DexaDays-2 study, because the questionnaires used in that study are validated only for those ages. Children who reach the age of 3 years during maintenance therapy and are still due to receive 5 dexamethasone courses after their birthday (at least 15 weeks before the end of therapy) are also eligible. Exclusion criteria of that study are anticipated compliance problems, underlying conditions that affect the absorption of oral medication, uncontrolled infections, or any other complications that may interfere with administering dexamethasone treatment, insufficient command of the Dutch language, preexisting mental retardation, and hydrocortisone or risperidone use during the invitation to participate.

**Figure 1:**
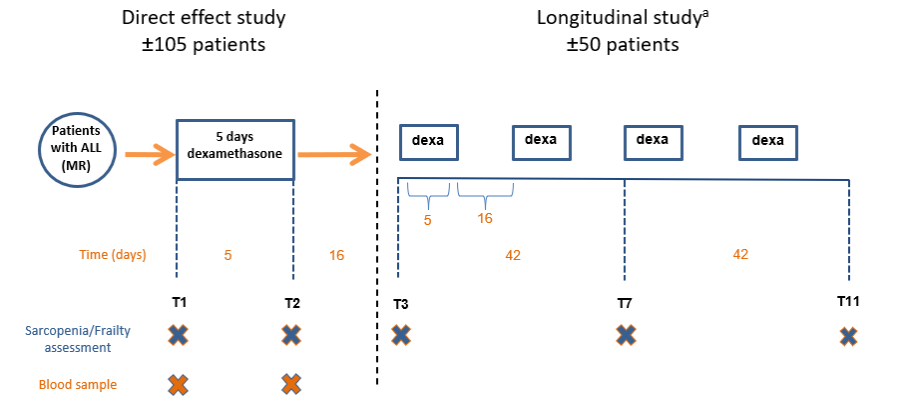
Timeline for sarcopenia and physical frailty assessment during the study. ^a^Patients continue with this part of the study when they have significant dexamethasone-induced behavioral problems, based on the study design of the DexaDays-2 study. ALL: acute lymphoblastic leukemia; MR: medium risk; Dexa: dexamethasone.

Our study on sarcopenia and frailty is being pursued within the framework of the aforementioned DexaDays-2 study. The latter consists of 2 parts: an identification study (including 2 timepoints: T1-T2) and a randomized controlled trial (T3-T11). The prospective observations of the current study are performed at a subset of these time points (Figure 1) during 15 weeks of the maintenance phase of ALL therapy.

A total of 105 patients will undergo a physical frailty assessment before (T1) and after 5 days (T2) of dexamethasone administration (6 mg/m^2^ per day in 3 dosages). A subset of 50 children will be assessed at 3 additional timepoints (T3, T7, and T11), to observe the process of sarcopenia and physical frailty longitudinally. This subset comprised children participating in the DexaDays-2 randomized controlled trial based on the severity of their neurobehavioral problems. These 50 patients will receive physiological dosages of hydrocortisone or placebo during a 5-day dexamethasone treatment, and the cross-over will take place after 2 courses of dexamethasone treatment. The sample size is based on the DexaDays-2 study power calculation [40].

### Ethics Approval

The study was approved by the Medical Ethics Committees of the Erasmus Medical Center Rotterdam (reference number: NL62388.078.174).

### Outcome Definitions: Sarcopenia and Physical Frailty

#### Sarcopenia

We will use the most widely cited definition of sarcopenia as proposed by the European Working Group on Sarcopenia [19,39], that is, the combination of low muscle strength or function, and impaired muscle mass [19]. For decades, the term sarcopenia had been used to describe muscle loss alone without reference to function. However, recent updates and consensus definitions state the importance of including muscle function in the concept of sarcopenia [39]. Therefore, in this study, sarcopenia is defined as a combination of impaired muscle mass and low muscle strength.

**Figure 2:**
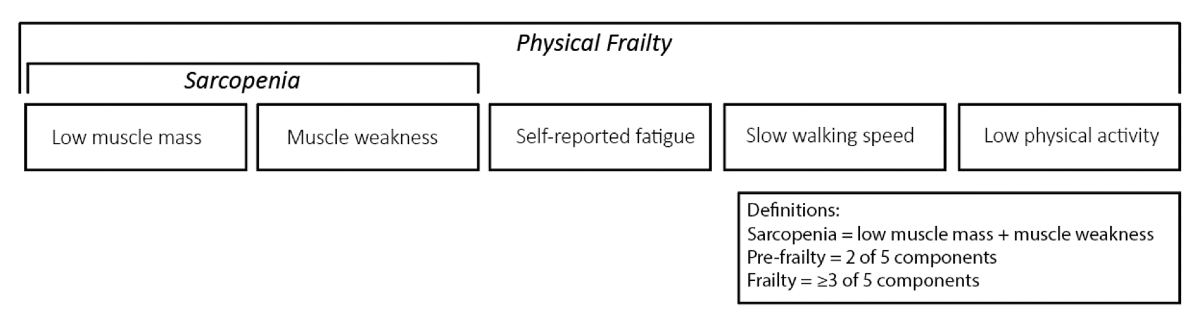
Physical frailty and sarcopenia definitions and the individual components.

These components are also separately included in the frailty definition (Table 1, Figure 2).

**Table 1:** Short overview of definition and measurements used to assess sarcopenia in children with ALL^a^.

EWGSOP^b^ sarcopenia definition	Concept used in previous pediatric ALL studies	Method used in our study
Loss of muscle mass	Psoas muscle area loss evaluated using computed tomography [20]. Skeletal muscle mass or lean body mass evaluated using dual-energy X-ray absorptiometry [4,21].	Skeletal muscle mass evaluation by bioimpedance analysis.
Muscle weakness	N/A^c^	Handgrip strength evaluation using handheld dynamometry.

^a^ALL: acute lymphoblastic leukemia.

^b^EWGSOP: European Working Group on Sarcopenia in Older People.

^c^NA: not applicable.

#### Physical Frailty

In accordance with the original definition of Fried et al [23] and previous clinical frailty studies in childhood cancer survivors, we will define frailty using the following components: low muscle mass, muscle weakness, self-reported fatigue, slow walking speed, and low physical activity [24,25]. Prefrailty and frailty will be defined, respectively, by the presence of 2, or 3 or more of the 5 components [22]. Assessments are excluded if 3 or more components are missing.

The outcome measures to examine sarcopenia and physical frailty components are selected based on suitability for children with an age range of 3-18 years (Table 2).

**Table 2:** Short overview of measurements used to assess frailty in childhood cancer survivors and in this study.

Frailty components	Concept used in CCS^a^	Method used in the current study in children with ALL^b^
Shrinking/weight loss	Lean muscle mass evaluated using DXA^c^ [24,25].	Skeletal muscle mass evaluation by bioimpedance analysis.
Weakness	Handgrip strength evaluated using handheld dynamometry [24,25].	Handgrip strength evaluation using handheld dynamometry.
Exhaustion	Self-reported exhaustion assessed using the SF-36^d^ [25] or during a semistructured interview [24].	Self-reported fatigue assessment using PedsQL-MFS^e^.
Slowness	Slow walking speed evaluated based on cut-off points for 15 ft [25], or by using the 6-Minute Walk Test [24].	Slow walking speed evaluation using the Timed Up and Go Test.
Low physical activity	Energy expenditure during leisure time physical activity based on the NHANES^f^ Physical Activity [25] and based on frequency and duration per week derived from a semistructured interview [24].	Energy expenditure evaluation based on physical activity questionnaires.

^a^CCS: childhood cancer survivors.

^b^ALL: acute lymphoblastic leukemia.

^c^DXA: dual x-ray absorptiometry.

^d^SF-36: 36-item Short Form Survey.

^e^PedsQL-MFS: Pediatric Quality of Life-Multidimensional Fatigue Scale.

^f^NHANES: National Health and Nutrition Examination Survey.

#### Skeletal Muscle Mass

Total body SMM will be measured using multifrequency segmental bioimpedance analysis (Tanita MC-780; Tanita Corporation) [41]. The measurement procedure requires the child to stand in bare feet on the analyzer and to hold a pair of handgrips, 1 in each hand for approximately 15 seconds. Subsequently, the Skeletal Muscle Index (SMI) will be calculated by dividing the individual SMM (kilogram) by height (m^2^; ie, SMI=SMM/height). The Tanita device has shown excellent test-retest reliability [42]. High significant correlations (correlation coefficients ≥0.85) were shown for body composition values in children and adolescents, between bioimpedance analysis and dual-energy X-ray absorptiometry (which is the golden standard for the measurement of muscle mass) [43].

#### Muscle Strength

Handgrip strength (kilogram) will be measured in sitting position with the elbow flexed at 90° using a hydraulic Jamar handheld dynamometer (Sammons Preston). During the measurement the child will be verbally encouraged to achieve a maximum performance. For both the dominant and nondominant hand the mean score of 3 repeats will be calculated. Raw results will be compared with population-based age- and sex-specific reference values [44]. Handgrip dynamometry showed good validity (intraclass correlation coefficients [ICCs] 0.73-0.91) with high reproducibility in children from the age of 4 years and has excellent test-retest reliability (ICC 0.91-0.93) [45,46], and the measurement was feasible in children with leukemia [47].

#### Fatigue

Fatigue-related complaints will be assessed using the validated Dutch version of the Pediatric Quality of Life Inventory (PedsQL)-Multidimensional Fatigue Scale (MFS) [48,49]. This questionnaire consists of 3 scales: General Fatigue, Sleep/Rest Fatigue, and Cognitive Fatigue, resulting in subscores and in a total fatigue score. We will use parent proxy reports for children aged 2-4, 5-7, 8-12, and 13-18 years, as well as self-report versions for children aged 8-12 and 13-18 years. The results of PedsQL-MFS will be compared with Dutch normative values from the general population [48]. The internal consistency of the Dutch version of the PedsQL-MFS has been reported as satisfactory (Cronbach coefficient α >.70), test-retest reliability was good (ICC 0.68-0.84), and the interobserver reliability varied from moderate to excellent (ICC 0.56-0.93) [48]. The original version of PedsQL-MFS has been validated in patients with pediatric cancer, 50% of whom had ALL [49], and the Dutch version has been used previously in studies in children with ALL [28,50].

#### Walking Speed

The TUG will be used to asses walking speed. A chair, without arm rests, allowing the child to sit with his feet flat on the floor and his hip and knees flexed at 90° will be used. The chair will be positioned at a 3-m distance from a wall. The child will be asked to get up from the sitting position and walk “as fast as he can, without running” to the wall and touch a self-chosen picture on the wall, turn around without using the wall for support, walk back to the chair, and sit down. During the test verbal instructions will be repeated and encouragements are made. Time is recorded from the “go” cue to when the child is sitting down in the chair. The mean time of 3 trials will be considered as the test result [51]. The results of the test will be compared with age-specific reference values [52]. The TUG has shown excellent test-retest reliability (ICC 0.80-0.98) and interobserver reliability (ICCs 0.86 to 0.99) in the pediatric population [53], and the measurement was feasible in children with leukemia [47].

#### Physical Activity

Physical activity will be estimated using parent and self-reported questionnaires. We will use questionnaires generated in a Dutch population–based prospective cohort study investigating the development of a cohort of newborn children until young adulthood [54]. We will use parent proxy-reported versions for children aged 3-11 years, and child-reported versions for children aged 9-11 years [55,56]. These questionnaires comprise questions regarding frequency and duration of outdoor playing, sports participation, and active transport to/from school, as well as sedentary behavior such as watching television and computer use. Children aged 12-18 years will be asked to fill in the modified Baecke questionnaire, which consists of 3 components: school activity, sports activity, and leisure activity [57]. The results of the physical activity questionnaires (type of activity, frequency, and duration) will be compared with the Youth Compendium of Physical Activities [58]. The age-specific metabolic equivalent of the specific activity (either leisure or sports) will be used to estimate the energy expenditure in calories of an individual participant.

In addition, we hypothesized that generalized muscle weakness might be better expressed in a functional performance test, which is currently not a part of the frailty assessments. To explore the potential value of a functional muscle strength measurement in the concept of sarcopenia and frailty, we will use the “Time to Rise From the Floor Test” (TRF) [59]. The child will be asked to sit in the cross-legged position on the floor and to get up as fast as possible. The TRF will be performed 2 times, and for both performances, the quantitative performance (time in seconds) and a quality grade will be scored. We will use the “Gowers maneuver” as a quality performance by grading the amount of support needed to rise [60]. This is a standardized method to quantify on a 1-7 scale, where 1 means normal rising and 7 means unable to rise (Table 3).

All assessments will be performed by a pediatric physiotherapist (EV) or a trained medical doctor (AvH).

**Table 3:** Gowers maneuver grade: to quantify the ability to rise from the floor^a^.

Performance	Grade
Normal rising	1
Butt-first maneuver, 1 hand on floor	2
Butt-first maneuver, 2 hands on floor	3
Unilateral hand support on thigh	4
Bilateral hand support on thighs	5
Arises only with aid of an object (table, chair)	6
Unable to arise	7

^a^Standardized method to quantify the quality of rising by grading the amount of support needed to rise.

### Potential Determinants

We will explore the following potential determinants for the components of sarcopenia and physical frailty: sex, age, weight Z-scores at diagnosis, time since the start of treatment, registered toxicity and serious adverse events in induction therapy, cumulative vincristine dosage, dexamethasone pharmacokinetics, and carrier of relevant genetic variants (candidate single-nucleotide polymorphisms). Information regarding these factors will be extracted from the electronic patient files or is collected in the DexaDays-2 study. Dexamethasone kinetics are measured through peak levels (2-3 hours after the first dexamethasone administration on day 1 of the dexamethasone course) and trough levels (measured on day 6, at least 12 hours after the last dexamethasone dose administered on the previous evening). A peripheral blood sample to extract germline DNA, for evaluation of carrier status of relevant candidate single-nucleotide polymorphisms related to sarcopenia and physical frailty, is taken on T1 as part of the DexaDays-2 study. As a complete array (Illumina GSA) will be run, we will be able to select the most relevant specific additional single-nucleotide polymorphisms of interest, based on evidence from the most recent literature (Figure 1).

### Statistical Analysis

The results of the physical frailty assessments, that is, SMI, handgrip strength, self-reported fatigue, walking speed, and physical activity (component scores), will be reported as means and SDs, or as median and interquartile ranges if data are non-normally distributed. The frequency of sarcopenia and physical frailty, as well as the individual components at all timepoints will be reported in percentages and schematically visualized. A correlation matrix will be built to explore coherence between the additional measure TRF and the other frailty components.

To evaluate the potential change in frailty components after 5 days of dexamethasone administration, a paired Student *t* test or Mann-Whitney *U* test will be used depending on the distribution of the data.

To evaluate whether the mean scores from the frailty components change between T1, T3, T7, and T11, 1-way ANOVA will be employed.

To study the effect of dexamethasone administration at T1, T3, T7, and prior to T11 on sarcopenia, physical frailty, and each individual components, generalized mixed models will be estimated. These models incorporate correlations between repeated responses on the same individual. Patient-specific random intercept, age, weight, time since the start of therapy, and cumulative vincristine dosage will be included in the model.

Multivariable linear regression model will be estimated to investigate associations between the potential determinants (patient- and treatment-related factors, pharmacokinetics, and genetics) and the assessment scores at T1 and T2 (SMI, handgrip strength, self-reported fatigue, walking speed, TRF, and physical activity). Results will be presented as regression coefficients along with 95% CI.

Multivariable logistic regression models will be estimated to explore associations between the aforementioned potential determinants and the occurrence of sarcopenia and physical frailty at T1 and T2. Odds ratios along with 95% CIs will be estimated.

Statistical analyses will be performed using software packages R Statistics (version 1.0.143; R Foundation) and SPSS (version 26.0.0.1) for Windows (IBM).

## Results

Patient accrual started in 2018 and was finalized in spring 2021. A total of 105 children undergoing ALL therapy will participate in this study. From autumn 2021 statistical analyses will be performed.

## Discussion

This paper describes the design of the first study on sarcopenia and physical frailty in children during maintenance therapy for ALL. The results of this study will provide information and create awareness on the magnitude and severity of sarcopenia and physical frailty in this specific group of children, which may help us understand which factors are associated with the large variations in physical ability between children receiving similar treatments. With this knowledge we may be able to identify physically vulnerable children at an earlier stage. This is important because being vulnerable to sarcopenia or frailty can complicate continuation of ALL therapy, independence in physical functioning, reaching motor milestones, and participation in daily life activities.

In this study we will assess sarcopenia involving a combination of low muscle mass *and* low muscle strength for the first time in patients with ALL. The SMM will be estimated using the Tanita MC-780 multifrequency segmental body composition analyzer, which is a validated, reliable, low-cost, fast, and non-invasive device to estimate body composition in the pediatric population [43].

None of the previous studies in children with ALL concerning muscle mass loss incorporated functional muscle strength assessments. We expect this to be of additional value because recent updates and consensus definitions state the importance of including muscle function in the concept of sarcopenia [61], as reduced muscle mass in combination with normal muscle strength may suggest malnutrition rather than sarcopenia [62]. Besides, in children, impaired muscle function directly influences motor development and is therefore relevant [63].

To explore this aspect further, we added a functional strength measurement to the assessment: the TRF. Although the handgrip dynamometer is a reliable instrument to measure handgrip strength in children [46], this may not be the first sign of reduction of muscle strength. We suspect that a generalized reduction in muscle strength (such as in sarcopenia and physical frailty) might be better shown by a functional performance test. The time and degree of support needed to rise from the floor are standardized measures to quantify deterioration and are associated with walking ability in children with muscular dystrophy [64].

The results of this study will provide knowledge about the effect of treatment with high doses of dexamethasone on muscle loss and other components of physical frailty, and therefore insights into the severity and risks of these side effects. Furthermore, through exploring potential determinants that could influence the occurrence of the sarcopenia or frailty, we might be able to identify children at risk for substantial problems at an earlier stage. As a result, we might be able to start targeted interventions and clinical studies on reducing the dexamethasone-induced components of physical frailty with, for example, nutrition and exercise.

This study has a number of strong points. First, because care for children with ALL in the Netherlands is centralized, a national cohort of Dutch children can be screened on eligibility for this study, rendering a large and hopefully unbiased population. Second, all children will have a physical frailty assessment by a skilled pediatric physiotherapist or a trained medical doctor, which benefits the validity and reliability of the performed physical assessment. Third, the burden of the study will be minimal because the assessments are performed during maintenance therapy of ALL, in which children experience less toxicities, fewer hospital admissions, and therapy is mainly administered at home and in the outpatient clinic. Fourth, we selected sarcopenia and frailty endpoints in accordance with previous research and the official definitions. Furthermore, we added 1 functional strength measurement, the TRF, based on expert opinion and on particular feasibility in children with ALL.

Some possible study limitations have to be taken into account as well. We will only include children participating in the DexaDays-2 study, which could potentially lead to selection bias, as patients who experience dexamethasone-induced neurobehavioral problems could be more motivated to participate because in that trial they will receive a drug to potentially reduce these problems. Furthermore, the subset of 50 children that will be measured longitudinally comprises children participating in the DexaDays-2 randomized controlled trial based on the severity of their neurobehavioral problems. These patients will receive physiological dosages of hydrocortisone or placebo during a 5-day dexamethasone treatment, and the cross-over will take place after 2 courses of dexamethasone treatment. We do not know if this affects the occurrence of sarcopenia and physical frailty in this cohort, for example, whether children with dexamethasone-induced clinically relevant neurobehavioral problems also show more physical side effects. Besides, within the DexaDays-2 study patients aged 3-18 years were selected, which complicated selecting outcome measures suitable for all participants. We succeeded in selecting measurements that have previously been used and validated in pediatric populations indicating their usefulness; however, with the exception of the PedsQL-MFS, none of the measurements has been validated in pediatric oncology patients.

In conclusion, this study is designed to determine the occurrence and severity of sarcopenia and frailty components in children during the maintenance phase of ALL therapy, and to determine the effects of administration of high doses of dexamethasone on physical vulnerability. With this study we aim to create awareness about these potential risks in children with ALL, as well as expanding our knowledge for reducing further side effects.

## Abbreviations

ALL: acute lymphoblastic leukemia
CTCAE: Common Terminology Criteria for Adverse Events
DCOG: Dutch Childhood Oncology Group
ICC: intraclass correlation coefficient
PedsQL-MFS: Pediatric Quality of Life Inventory-Multidimensional Fatigue Scale
SMI: Skeletal Muscle Index
SMM: skeletal muscle mass
TRF: time to rise from the floor
TUG: Timed Up and Go Test
